# Supplementary material for: Effect of Immobilization of Phenolic Antioxidant on Thermo-Oxidative Stability and Aging of Poly(1-trimethylsilyl-1-propyne) in View of Membrane Application
Source: Polymers (Basel). 2022 Jan 24;14(3):462. doi: 10.3390/polym14030462 (PMC8838476; doi:10.3390/polym14030462)
Supplement: Supplementary file 1 [file polymers-14-00462-s001.zip › polymers-1521891-supplementary.pdf]

Supporting information for the manuscript:

Effect of Immobilization of Phenolic Antioxidant on Thermo-Oxidative Stability and Aging of Poly(1-trimethylsilyl-1-Propyne) in View of Membrane Application

Sergey Shishatskiy [1,*], Vladimir Makrushin [2], Ivan Levin [2], Petra Merten [1], Samira Matson [2,*] and Valeriy Khotimskiy [2,*]

[1] Helmholtz-Zentrum Hereon, Institute of Membrane Research, Max-Planck-Str. 1, 21502 Geesthacht, Germany; petra.merten@hereon.de

[2] A.V. Topchiev Institute of Petrochemical Synthesis of Russian Academy of Sciences, Leninsky Prospect 29, 119991 Moscow, Russia; makrushin@ips.ac.ru (V.M.); levin@ips.ac.ru (I.L.)

* Correspondence: sergey.shishatskiy@hereon.de (S.S.); matson@ips.ac.ru (S.M.); hotimsky@ips.ac.ru (V.K.); Tel.: +49-4152-87-2467 (S.S.); +7-495-647-5927 (ext. 214) (S.M.)

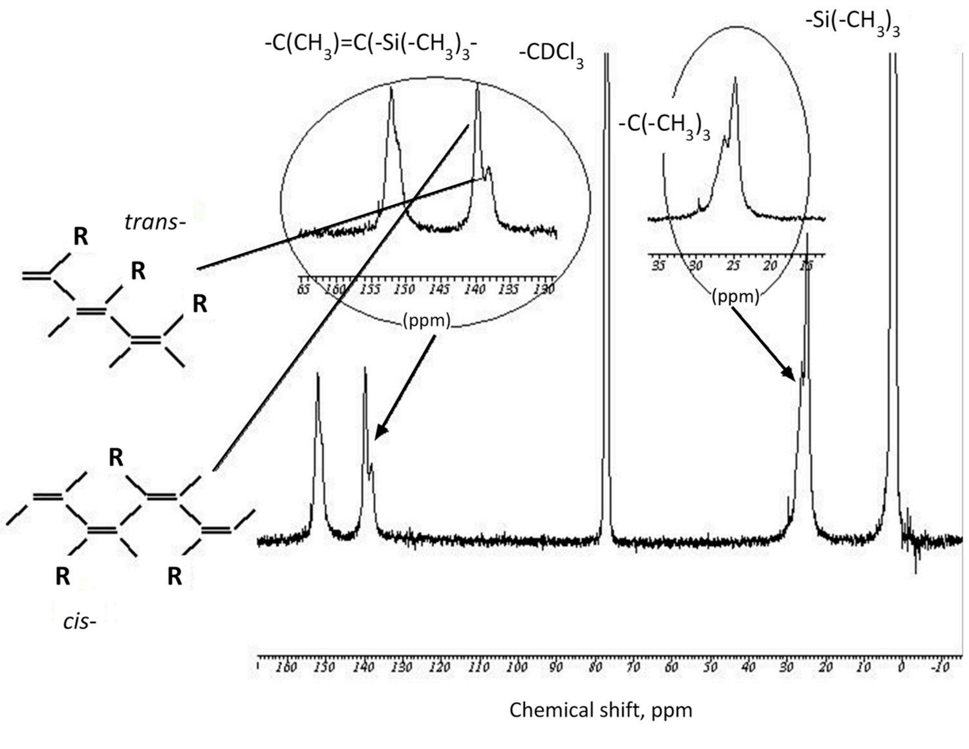

Figure S1. ^{13}C-NMR spectra of PTMSP used for the quantitative determination of *cis*- and *trans*-units ratio in polymer samples.
